# In rats fed high-energy diets, taste, rather than fat content, is the key factor increasing food intake: a comparison of a cafeteria and a lipid-supplemented standard diet

**DOI:** 10.7717/peerj.3697

**Published:** 2017-09-13

**Authors:** Laia Oliva, Tània Aranda, Giada Caviola, Anna Fernández-Bernal, Marià Alemany, José Antonio Fernández-López, Xavier Remesar

**Affiliations:** 1 Department of Biochemistry and Molecular Biomedicine, University of Barcelona, Faculty of Biology, Barcelona, Spain; 2 Institute of Biomedicine, University of Barcelona, Barcelona, Spain; 3 CIBER OBN, Centro de Investigaciones Biomédicas en Red, Barcelona, Spain

**Keywords:** High-fat diet, Cafeteria diet, Taste, Food intake, Rat

## Abstract

**Background:**

Food selection and ingestion both in humans and rodents, often is a critical factor in determining excess energy intake and its related disorders.

**Methods:**

Two different concepts of high-fat diets were tested for their obesogenic effects in rats; in both cases, lipids constituted about 40% of their energy intake. The main difference with controls fed standard lab chow, was, precisely, the lipid content. Cafeteria diets (K) were self-selected diets devised to be desirable to the rats, mainly because of its diverse mix of tastes, particularly salty and sweet. This diet was compared with another, more classical high-fat (HF) diet, devised not to be as tasty as K, and prepared by supplementing standard chow pellets with fat. We also analysed the influence of sex on the effects of the diets.

**Results:**

K rats grew faster because of a high lipid, sugar and protein intake, especially the males, while females showed lower weight but higher proportion of body lipid. In contrast, the weight of HF groups were not different from controls. Individual nutrient’s intake were analysed, and we found that K rats ingested large amounts of both disaccharides and salt, with scant differences of other nutrients’ proportion between the three groups. The results suggest that the key differential factor of the diet eliciting excess energy intake was the massive presence of sweet and salty tasting food.

**Conclusions:**

The significant presence of sugar and salt appears as a powerful inducer of excess food intake, more effective than a simple (albeit large) increase in the diet’s lipid content. These effects appeared already after a relatively short treatment. The differential effects of sex agree with their different hedonic and obesogenic response to diet.

## Introduction

Fat intake is correlated with weight gain and increased body fat content (Rothwell & Stock, 1984). The use of different diets with high energy content has been widely used to determine the conditions eliciting overweight or obesity (Hariri & Thibault, 2010). Obesogenic diets have been used to provoke important changes in rodents, especially those related to adipose tissue growth and, as a consequence, their increased involvement in carbohydrate and lipid metabolism (Peckham, Entenman & Carroll, 1977; Archer et al., 2007). A wide variety of high-energy diets have been used, in which the high lipid content is the common link, thus indicating that dietary fat is a critical factor for fat accumulation (Buettner, Schölmerich & Bollheimer, 2007). However, there is considerable variability in the composition of the high-fat diets (HF) used in different obesity models, since the proportion of lipids and their fatty acid composition make these diets highly heterogeneous (Oakes et al., 1997; Briaud et al., 2002), by far, different from controls on standard chow. In addition, most HF diets contain high fructose or sucrose to enhance their obesogenic effects. They are often simplified (standardized), using a single fat and/or protein source (Sato et al., 2010). The metabolic effects of these diets are variable depending on several factors, such as the age of the animals (Sclafani & Gorman, 1977), the duration course of the intervention (Schemmel, Mickelsen & Tolgay, 1969), the energy density of the diet and, especially, sex (Agnelli et al., 2016).

The cafeteria diet is a palatable food diet model in which the range (and variety of tastes and texture) of the foods offered induce a marked hedonic-driven increase in food (and thus energy) consumption (Sclafani & Springer, 1976; Prats et al., 1989). This consequent excess of energy intake results in the excessive accrual of fat, despite the homoeostatic response to lower food intake and increased thermogenesis (Rothwell, Saville & Stock, 1982). Cafeteria diets have been widely used to fatten rats, but a number of Authors tend to consider that the variability attributed to self-selection by taste may be a serious handicap of this model (Moore, 1987). Cafeteria diets are very effective creating a model of metabolic syndrome (Gomez-Smith et al., 2016), which can cause oxidative damage in adipose tissues (Johnson et al., 2016), although it also lowers the anxiety of rats (Pini et al., 2016) attenuating their response to stress (Zeeni et al., 2015) because of the “comfort food effect” (Ortolani et al., 2011). On the other hand, the analysis of what food items were selected by the rats is laborious, but the results obtained are precise, and may allow us to measure the change with exposure time during different phases of development (Prats et al., 1989; Lladó et al., 1995). The fact remains that cafeteria diets are more obesogenic than standard high-lipid diets with equivalent energy content; despite the variability associated to selection, the actual precise and statistically invariable nutrient intake (Esteve et al., 1992a) overcomes the rat strict energy intake control. The consequence is a higher lipid deposition, metabolic change and inflammation (Rafecas et al., 1992; Romero et al., 2014).

A critical difference between cafeteria diet and “fixed composition” HF diets, in spite of their equivalence in lipid-derived energy, is the (constant) abundance of at least two key tasty components, salt and sugar, which enhance the appetite for food, and consequently increase energy intake (Tordoff & Reed, 1991; Breslin, Spector & Grill, 1995). A number of HF diets are also additionally sugar-laden, being very effective in eliciting fat deposition (Sato et al., 2010).

In this study, we used a model of HF diet matched in composition (except fat) to the standard rat chow. We used coconut oil (rich in saturated fat), that has a moderate obesogenic capacity (Buettner et al., 2006; Hariri & Thibault, 2010) when not supplemented with sucrose. This fat content was selected to coincide with the known “usual” percentage of fat self-selected by rats using our simplified cafeteria diet model (ca. 40%) (Esteve et al., 1992a; Rafecas et al., 1993). The proportion of essential lipids in control diet and our HF diet was the same (i.e., PUFA), being the difference essentially C12–C16 (saturated and monounsaturated) fatty acids. The uniformity in the energy derived from lipids between HF diet *vs.* cafeteria, and the equivalence in everything else except lipid between control diet and HF diet allowed us to establish comparisons based on comparable facts, a point that, as far as we know, has not been previously attempted.

We tried to analyse the influence of tasty food (and consequent activation of the satisfaction circuits) on body energy balance and the known metabolic alterations induced by hyperlipidic diets. Our aim was to determine whether a relatively short treatment is sufficient to show the hedonic response to diet on increased food (and energy) consumption and lipid deposition, taking into account the influence of sex.

## Materials & Methods

### Diets

Standard diet (C) (Teklad 2014, Teklad diets, Madison WI, USA) contained 20% of digestible energy derived from protein, 13% from lipids, and 67% from carbohydrates (including 0.10% oligosaccharides). This diet essentially contained plant-derived foods.

The high-fat diet (HF) was prepared by the addition of coconut oil (Escuder, Rubí, Spain) to coarsely ground standard chow. The mix, containing 33 parts (by weight) of standard chow, 4 of coconut oil, and 16 parts of water, was thoroughly kneaded, to form a rough paste which was extruded using cut-end syringes to form 1 × 6 cm cylindrical pellets which were dried at 40 °C for 24 h. This diet contained 14.5% of digestible energy derived from protein, 37.0% from lipids, and 48.5% from carbohydrates. Aversion tests to this diet gave negative results, i.e., not different from control diet.

The simplified cafeteria diet (K) was formed by excess offering of the standard chow pellets, plain cookies spread with liver pâté, bacon, water and milk, which was supplemented with 300 g/L sucrose and 30 g/L of a mineral and vitamin supplement (Meritene, Nestlé, Esplugues, Spain) (Esteve et al., 1992a; Rafecas et al., 1993). All components were kept fresh (i.e., renewed daily). From the analysis (*a posteriori*) of the ingested items and diet composition, we calculated that approximately 41% of ingested energy was derived from lipids, 12% from protein, and 47% of energy was derived from carbohydrates (23% oligosaccharides and 24% starches), with fair uniformity between sexes (*p* > 0.05).

Table 1 presents the composition of the diets used. For K rats we used the actual food consumption data. Both, crude and digestible energy content per g were higher in the HF diet, since it contains more energy per g than the C and K diets. Cafeteria diet had the lowest crude energy value because of its low content of fibre, although its digestible energy was similar to that of control chow. The diet fat content was essentially the same for K and HF diets, i.e., 3-fold higher than that of C diet.

**Table 1:** Diet composition.

	Standard diet (C)	High-fat diet (HF)	Cafeteria diet^*^ (K)
g/kg			
Protein	143	116	91.6 ± 2.1
Fat	40	134	139 ± 3.5
Carbohydrate	480	390	361 ± 2.9
of which sugars	<1	<1	153 ± 7.4
Fibre	181	146	25.7 ± 4.8
Minerals	47	38	11.4 ± 0.6
Crude energy content (kJ/g)	16.5	18.8	12.4 ± 0.15
Digestible energy content (kJ/g)	12.1	14.6	12.0 ± 0.14

**Notes.**

* The data for K were the mean ± SEM of six pairs of rats; no significant differences in the proportions of food eaten were observed between sexes.

### Animals and experimental setup

All animal handling procedures and the experimental setup were carried out in accordance with the animal handling guidelines of the European, Spanish and Catalan Authorities. The Committee on Animal Experimentation of the University of Barcelona authorized the specific procedures used (# DAAM 6911).

Ten-week-old male and female Wistar rats (Janvier, Le-Genest-Saint-Isle, France) were used (*N* = 39). The animals were randomly divided in three groups (*n* = 6–8 for each sex) and were fed *ad libitum* for 30 days, either standard rat chow, oil-enriched rat chow (HF) or a simplified cafeteria diet (K). All animals had free access to water. They were housed (in same-sex pairs) in solid-bottom cages with wood shards as bedding material and were kept in a controlled environment (lights on from 08:00 to 20:00, temperature 21.5–22.5 °C, and 50–60% humidity). Body weight and food consumption were recorded daily. Calculation of ingested food in cafeteria diet fed rats was done as previously described by weighing the differences in food offered and debris left (Prats et al., 1989), correcting for dehydration.

On day 30, at the beginning of light cycle, the rats were anesthetized with isoflurane and then killed by exsanguination through the exposed aorta using a dry-heparinized syringe. Plasma was obtained by centrifugation and kept at −20 °C until processed. The carcass (and remaining blood and debris) were sealed in polyethylene bags, which were subsequently autoclaved at 120 °C for 2 h (Esteve et al., 1992b); the bag contents were weighed and then minced to a smooth paste with a blender (thus obtaining a total rat homogenate).

### Analytical procedures

Diet components were used for nitrogen, lipid and energy analyses. Nitrogen content was measured with a semi-automatic Kjeldahl procedure using a ProNitro S system (JP Selecta, Abrera, Spain), whereas lipid content was measured with a solvent extraction method (trichloromethane/methanol 2:1 v/v) (Folch, Lees & Sloane-Stanley, 1957). These procedures were also used for the determination of carcass lipid and protein content determination. The energy content of diet components and rat carcasses were determined using a bomb calorimeter (C7000, Ika, Staufen, Germany).

Glucose in plasma was measured under controlled conditions (15 min, 30 °C) with a glucose oxidase kit #11504 (Biosystems, Barcelona, Spain) supplemented with mutarotase (490 nkat/mL of reagent) (Calzyme, San Luis Obispo, CA, USA). Mutarotase was added to speed up epimerization equilibrium of α- and β-D-glucose and thus facilitate the oxidation of β-D-glucose by glucose oxidase (Miwa et al., 1972; Oliva et al., 2015). Other plasma parameters were measured with commercial kits; thus urea was measured with kit #11537, total cholesterol with kit #11505, creatinine with kit #11802 and triacylglycerols with kit #11528 (all from BioSystems, Barcelona, Spain). Lactate was measured with kit #1001330 (Spinreact, Sant Esteve d’en Bas, Spain) and non-esterified fatty acids with kit NEFA-HR (Wako, Neuss, Germany); 3-hydroxybutyrate and acetoacetate were estimated with a ketone bodies kit (Biosentec, Toulouse, France) based on 3-hydroxybutyrate dehydrogenase. Total plasma protein was measured using the Folin-phenol reagent (Lowry et al., 1951).

### Calculations and statistical procedures

Energy intake was calculated from daily food consumption converted with the energy equivalence of the different foods and components measured with the bomb calorimeter. Energy expenditure was calculated as previously described (Rothwell, Saville & Stock, 1982) from the difference between the ingested energy and the increase in body energy content of the animals. Energy content increase was estimated using reference data from our previous studies using rats of the same stock, age and sex (Romero et al., 2013; Romero et al., 2014). Sodium (salt) intake was calculated from food intake and the sodium content of the different food components used (Fernández-López et al., 1994).

Statistical comparisons were done with two-way ANOVA analyses (diet and time for weight changes, and sex and diet for the other data) and the *post hoc* Bonferroni test, using the Prism 5.0 program (GraphPad Software Inc, La Jolla CA, USA). Differences were considered significant when *p* value was <0.05.

## Results

**Figure 1:**
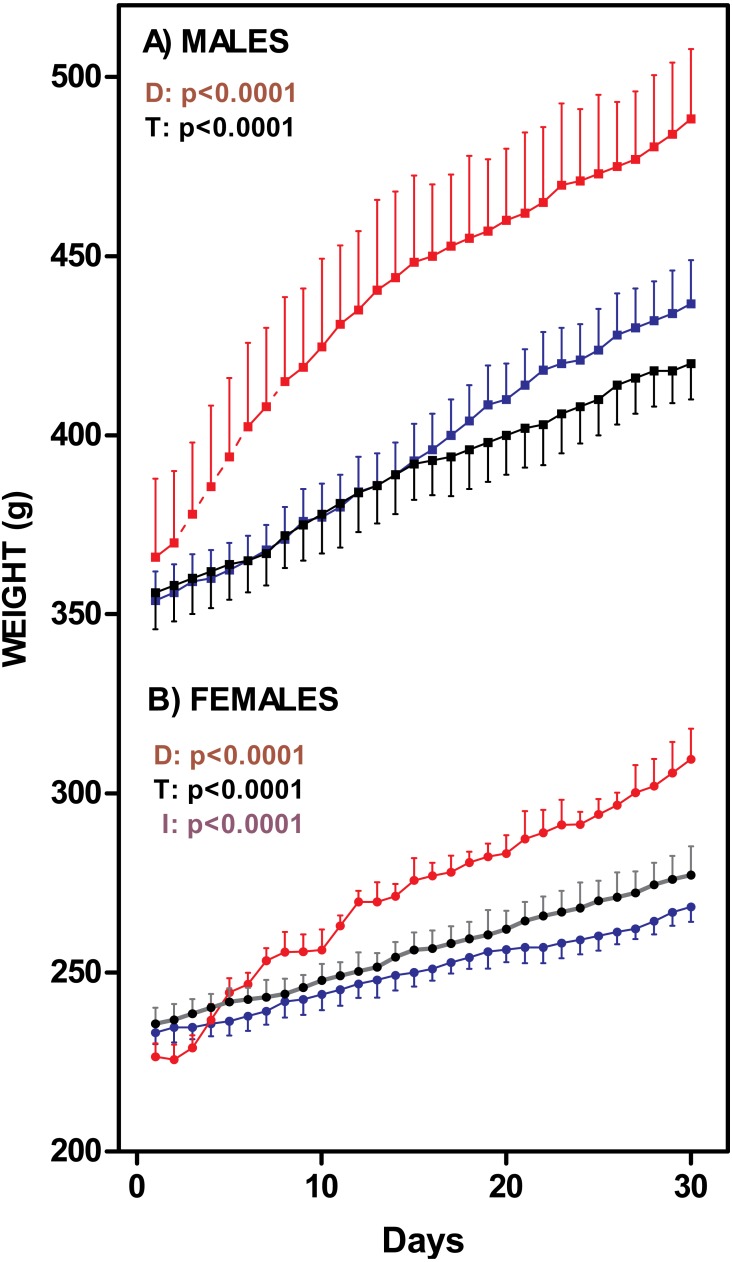
Rat weight changes through 30-days of dietary treatment. (A) represent males (squares) and (B) represent females (circles). Data are expressed as mean ± SEM of six to eight animals per group. Black: rats fed standard diet (C); Blue: rats treated with standard diet supplemented with fat (HF); Red: rats fed cafeteria diet (K). Statistical comparison were established by two-way ANOVA (T, time; D, diet; I, their interaction) and the Bonferroni *post-hoc* test (*p* < 0.05).

Figure 1 presents the changes on rat body weight after one-month of exposure to the diets. The males fed the cafeteria diet showed a significant weight gain (35%) with 1 month treatment; C and HF groups showed a similar, albeit lower, weight gain (18% and 22% respectively). The female K group showed the same pattern as males did (increase of 36%), but differences between K and C (16%) or HF (15%) groups were more marked than in males. There were no differences between C and HF groups. Nevertheless, K and C male weights were different from day 25 onwards. In females, the K group differed from HF from day 12 onwards, and the control group from day 19 onwards. Cafeteria-fed groups showed higher *in vivo* weight increases (males: 126 ± 3 g; females: 74 ± 7 g) than C (males: 79 ± 8 g; females: 40 ± 4 g) and HF (males: 83 ± 6 g; females: 28 ± 2 g) groups (Two-way ANOVA: Sex =*p* < 0.0001; Diet =*p* < 0.0001).

Table 2 shows the concentration of plasma metabolites. Female HF rats had lower glycaemia than C. When compared with controls, HF elicited a significantly higher lactate levels in both males and females. This HF diet also lowered cholesterol levels *vs.* controls irrespective of sex, but only males showed high triacylglycerols similar to those found in K. Compared with controls, the K males (but not females) showed higher free fatty acids. Urea levels were lower in K males *vs.* C, in contrast with females, which HF group also showed higher urea levels than C. Ketone bodies, especially 3-hydroxybutyrate levels, were affected by diet tending to show higher levels in the HF groups.

**Table 2:** Plasma parameters of rats fed Standard diet (C), High-fat diet (HF) or Cafeteria diet (K).

	Males			Females			*p*-value	
	C (*n* = 8)	HF (*n* = 6)	K (*n* = 7)	C (*n* = 6)	HF (*n* = 6)	K (*n* = 6)	D	S
Glucose (mM)	10.4 ± 0.33^A^	9.53 ± 0.47^A^	10.9 ± 0.64^A^	10.7 ± 0.63^a^	8.58 ± 0.21^b^	10.8 ± 0.56^a^	0.0029	ns
Lactate (mM)	2.17 ± 0.07^A^	4.67 ± 0.39^B^	2.71 ± 0.20^A^	2.11 ± 0.21^a^	3.91 ± 0.20^b^	2.55 ± 0.19^a^	<0.0001	ns
Cholesterol (mM)	2.57 ± 0.18^A^	1.82 ±0.02^B^	2.38 ± 0.12^A^	2.57 ± 0.09^a^	1.76 ± 0.19^b^	2.69 ± 0.12^a^	<0.0001	ns
Triacylglycerols (mM)	1.27 ± 0.07^A^	1.87 ± 0.12^B^	1.82 ± 0.21^B^	0.95 ± 0.10^a^	0.98 ± 0.16^a^	0.99 ± 0.09^a^	ns	<0.0001
Non-esterified fatty acids (mM)	0.32 ± 0.05^A^	0.44 ± 0.03^AB^	0.51 ± 0.06^B^	0.34 ± 0.07^a^	0.36 ± 0.02^a^	0.40 ± 0.06^a^	ns	ns
Total protein (g/L)	67.7 ± 0.68^A^	69.1 ± 0.64^A^	70.5 ± 1.72^A^	63.4 ± 1.92^a^	64.8 ± 1.78^a^	66.9 ± 1.27^a^	ns	0.0032
Urea (mM)	2.67 ± 0.21^A^	3.41 ± 0.05^A^	1.93 ± 0.18^B^	2.05 ± 0.13^a^	3.20 ± 0.37^b^	1.84 ± 0.21^a^	0.0001	ns
3-Hydroxybutyrate (µM)	30.2 ± 4.91^A^	50.5 ± 4.42^A^	30.8 ± 5.80^A^	45.3 ± 6.92^ab^	61.9 ± 10.9^a^	30.3 ± 6.83^b^	0.0028	ns
Acetoacetate (µM)	188 ± 43.1^A^	157 ± 31.1^A^	126 ± 16.1^A^	143 ± 61.0^a^	177 ± 29.8^a^	202 ± 51.8^a^	ns	ns

**Notes.**

Data expressed as mean ± SEM. Statistical analysis: two-way ANOVA, *p*-values for diet (D) or sex (S); *ns* = *p* > 0.05. No significant differences were found for the interaction between diet and sex. Bonferroni’s *post-hoc* test statistical significance, established at *p* < 0.05, is represented by different superscript letters.

**Figure 2:**
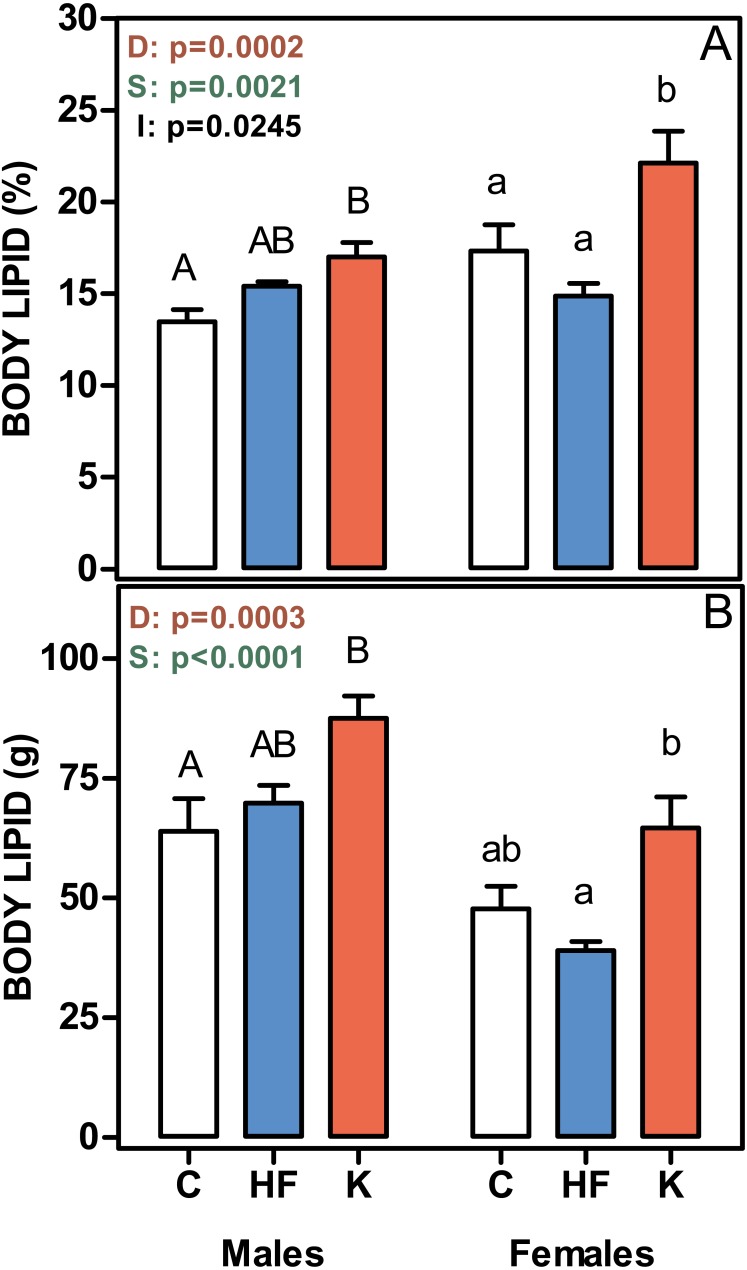
Body lipid content, expressed as a percentage of body weight, and in absolute values. (A) body lipid content as a percentage of body weight. (B) represent the total body lipid content (g). Data are the mean ± SEM of six to eight animals per group. White bars: standard diet (C); blue: high-fat diet (HF) and red: cafeteria diet (K). Statistical differences between groups: two-way ANOVA (D, diet; S, sex; I, their interaction). Bonferroni *post-hoc* test: different letters represent statistically significant (*p* < 0.05) differences between groups of the same sex.

Figure 2 shows that the percentage of body lipid was increased in both male and female cafeteria-fed rats, whereas there were no differences between the C and HF groups. The same pattern was observed when body lipid content was expressed in absolute values. Thus, body lipid was a main determinant of absolute body weight gain.

**Figure 3:**
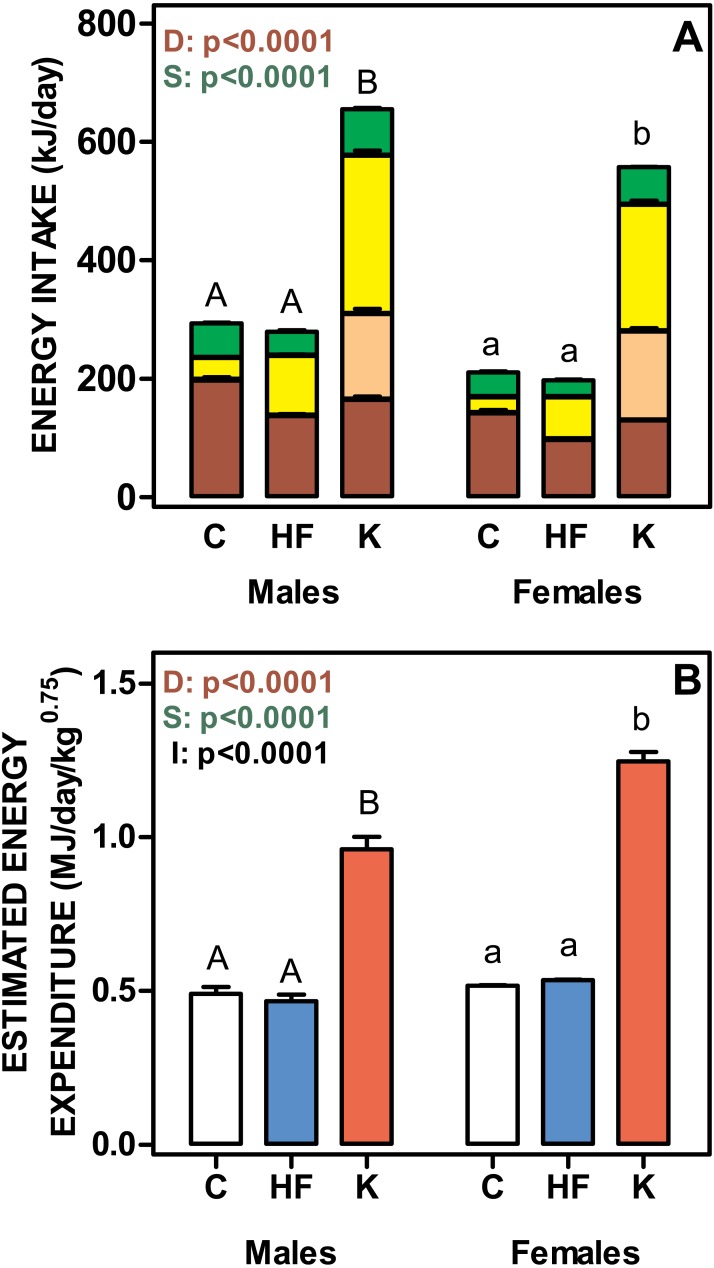
Total daily nutrient intake and estimated daily energy expenditure of rats treated for 30-days with standard, high-fat or cafeteria diets. (A) daily intake of protein, lipids, oligosaccharides and polysaccharides. Energy intake is expressed as kJ/day for each nutrient as stacked columns: brown bars represents polysaccharides; light brown bars oligosaccharides; yellow bars lipid and green bars protein. (B) estimated total daily energy expenditure expressed as MJ/day/weight^0.75^. White bars: standard diet (C); blue: high-fat diet (HF) and red: cafeteria diet (K). Data are the mean ± SEM of six to eight animals per group. Statistical significance of the differences were estimated for each nutrient group using two-way ANOVA (D, diet; S, sex; I, their interaction) and the Bonferroni *post-hoc* test showed differences between groups. Different letters represent statistically significant (*p* < 0.05) total energy intake/expenditure differences between groups of the same sex.

Figure 3 shows the daily energy intake and estimated energy expenditure of rats fed the three experimental diets. Cafeteria fed groups showed the highest values for both daily energy intake and energy expenditure. No differences were found between C and HF, in spite of the significantly lower polysaccharide and protein intake and higher lipid ingestion of the HF groups. The energy values for the different components were balanced, and thus the total energy intake was similar for C and HF groups. Cafeteria-fed rats showed significant increases in the energy intake derived from all diet components, especially for oligosaccharides, which represented 47 ± 2% of carbohydrate energy intake for males and 53 ± 2% for females (ns). Protein, lipid and polysaccharide intake showed different values (*p* < 0.0001) for diet and sex. Lipid and polysaccharide intake also showed statistically significant interaction between diet and sex (*p* = 0.0030).

**Figure 4:**
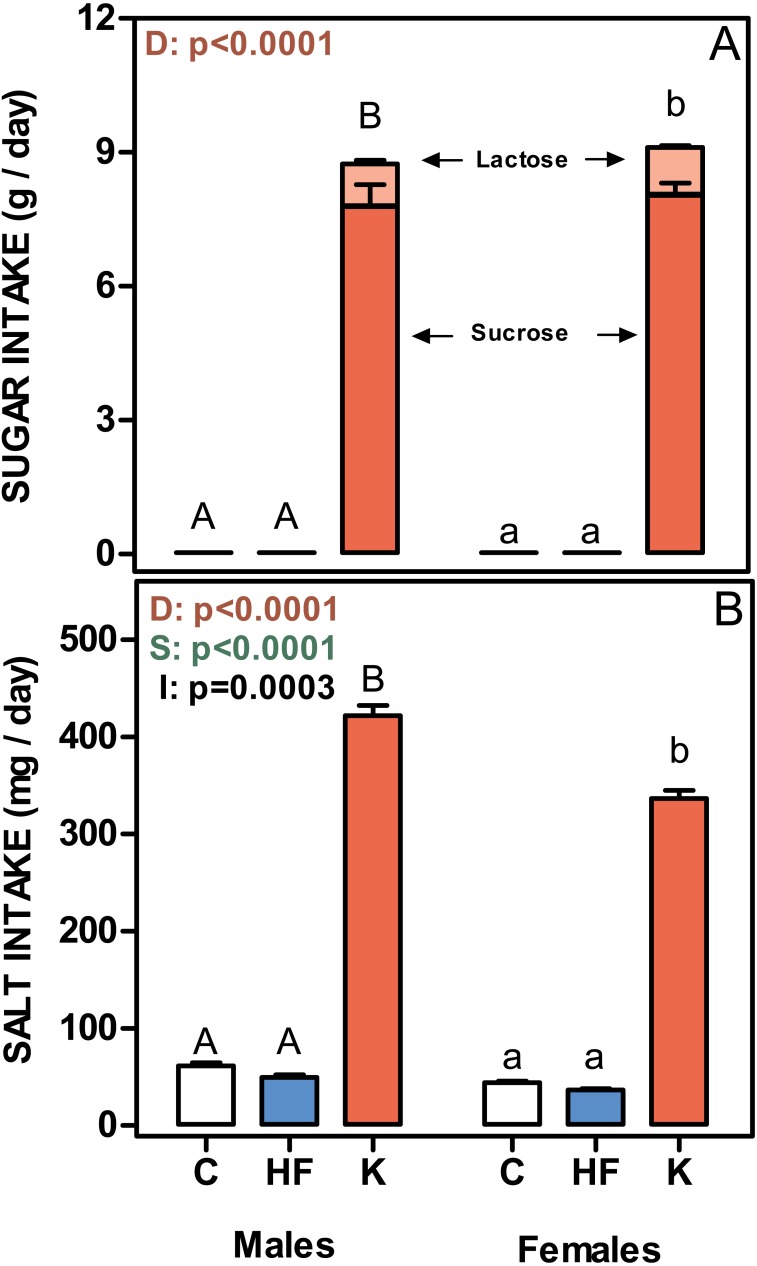
Sugar and salt intakes of rats treated for 30-days with standard, high-fat or cafeteria diets. (A) sugar intake. Values are given as g/day of lactose (light red) + sucrose (red). (B) Salt intake. Values are presented as mg of NaCl per day. Data are the mean ± SEM of six to eight animals per group. White bars: standard diet (C); blue: high-fat diet (HF) and red: cafeteria diet (K). Statistical differences between groups: two-way ANOVA (D, diet; S, sex; I, their interaction); Bonferroni *post-hoc* test: different letters represent statistically significant (*p* < 0.05) differences between groups of the same sex.

Figure 4 shows the mean daily rat intake of sugar and salt. The differences in sugar (either lactose or sucrose) intake were considerable, since C and HF intake (only sucrose) was very low compared with that ingested by the K groups. There were no differences between sexes. The daily salt intake was also higher in cafeteria groups (higher in males than in females), and a significant interaction with sex was observed. However, when expressed in mg/g of accrued weight, female rats ingested more salt than males (39 ± 0.7 in males and 56 ± 1.2 in females; *p* = 0.0061).

## Discussion

The main finding of this study is that, paradoxically, 30-day exposure to two types of high fat diet, with similar fat content but markedly different taste, texture and food variety, elicited widely different effects in body weight. The gain in weight shown by the HF-diet fed animals was similar to that of controls on fed standard food pellets, and agrees with data previously described for rats of the same-age kept on a standard diet (Buettner, Schölmerich & Bollheimer, 2007; Martire et al., 2013), although the results were also influenced by sex. The known obesogenic effects of cafeteria diets resulted in a significant increase in body weight in a relatively short-term (Romero et al., 2014). This increase was largely caused by the accumulation of fat, mainly in adipose tissue, although the increase in fat content is generalized to all tissues (Esteve et al., 1992a). Body lipid accrual was more marked in males. The absence of significant water retention again confirms that the main cause of weight increase was a consequence of the massive lipid accretion. Both high-lipid diets contained the same proportion of fat and had a similar proportion of the other macronutrients, but HF did not elicit an increase in body weight as K did. The difference was in the higher overall amount of energy ingested by the rats in the K group.

The differences in energy intake between HF and K groups were not caused by the dissimilar fibre content, since energy intake is a function of energy density irrespective of the presence of fibre content (Ramirez & Friedman, 1990). High fibre content induces a drastic reduction of food intake (and body weight) in rats previously fattened with high fat diet (Adam et al., 2016), probably as a consequence of lower diet energy density. The scant differences in digestible energy content between C and K, for instance, is an additional argument to assume that fibre has a minimal effect on food consumption in our model.

Tasty components of the diet have been considered as the main agents responsible of cafeteria diets overcoming the rat strict control on energy intake of cafeteria diets (Radcliffe & Webster, 1976; Mrosovsky & Powley, 1977), and also of decreasing their satiety threshold (Reichelt, Morris & Westbrook, 2014), even with relatively short periods of exposure. These effects may help explain the hyperphagia (causing the increased energy intake) observed in the rats fed a cafeteria diet, since its effects on appetite are mediated by a short-term increase in sympathetic activity (Muntzel et al., 2012). The effect of the high energy density of the diet, tending to decrease the overall food intake (Ramirez & Friedman, 1990), seems not to be effective in the K groups. Thus, the acknowledged taste components of cafeteria diet (essentially sugar and salt, i.e., sweet and salty) seem to be more effective agents acting on the control of appetite than the possible palatability of fats (and fatty acids) also present in the HF diet in amounts similar to those of cafeteria diet. This factor should be considered with the context of both the induction of food intake caused by variety (and novelty) of foods and tastes (Moore et al., 2013), which in part exploits the “explorative” drive shared by rats and humans. In addition, the intake of pleasing food (such as sweets), lowers the levels of anxiety (Faturi et al., 2010), and is used (by humans and experimental animals alike) as “comfort food” (Ortolani et al., 2011) to escape of conflict situations, or simply for pleasure (Pini et al., 2016).

The estimated values for energy expenditure and the percentage of body lipid content indicate that HF rats closely paralleled the energy balance of the control diet groups, and differ markedly from those fed K diet. The lower lipid storage in the HF rats, despite their high intake of lipids (largely consisting in saturated fatty acids and the PUFA from the standard diet from which HF diet was fabricated) suggests that in HF rats, dietary lipids were oxidized almost quantitatively. Their energy simply compensated the decreased carbohydrate utilization due to the compounded effect of its lower presence in the diet and lower food intake.

It must be taken into account that the lipids ingested were almost exclusively as acylglycerols, not free fatty acids, and thus it is unlikely that the actions on lingual fatty acid receptors (Mizushige, Inoue & Fushiki, 2007) could play a significant role in the taste of this diet.

Nevertheless, the greasy texture that lipid confer to high-fat diets seems to be attractive for rats (Hamilton, 1964) (as in humans (Kant et al., 2008)). Notwithstanding, our data showed that rats fed HF diet did not show a higher food intake than controls, which seems to just eliminate the “lipid taste” as a critical factor for hyperphagia. This conclusion may be an unexpected consequence of the HF diet formulation we used, being essentially the standard diet with added fat, and not a wholly different diet, formed by a few simple components (protein, starches, sugars and fats), as those commonly used for studies on obesity (Crescenzo et al., 2015).

Our data help clarify the situation, since they prove that fat (alone) could not be the key factor eliciting a higher food (energy) intake. The case in point being the sucrose-oil HF diets commonly used to induce obesity in rodents (Kanarek & Marks-Kaufman, 1979) even when coconut oil was used (Portillo et al., 1998; Ellis, Lake & Hoover-Plow, 2002). Probably, in these diets, the sugar plays a deeper effect on the obesogenic properties of diet than usually assumed (Sclafani, 1987). The significant increase in 3-hydroxybutyrate levels caused by diet (indicating active fatty acid disposal), especially marked in HF rats, may act also as a satiety signal (Scharrer, 1999), thus helping maintain food intake in an already relatively low setting. This was compounded mainly in females, by an efficient catabolic use of lipids.

The results obtained using this model, proved that fat alone was not the main inducer of hyperphagia. Consequently, we should determine what other dietary factors could justify the marked differences in food (and energy) intake between HF and K diets (sharing a similar proportion of dietary fat content). We postulate that this difference should be attributed to the massive intake of sugar and salt in addition to other psychological variables such as variety and comfort. These nutrients are present in relatively large proportions in all cafeteria diet formulations, and are often absent or in low proportion in most standard rodent diets, much closer to natural life conditions. Up to now, these components have received only scant attention as inducers of cafeteria diet-driven hyperphagia. Sugar (sweet taste) causes pleasurable sensations in rodents due to their oral sensory properties (Peciña, Smith & Berridge, 2006) that seek and stimulate the consumption of sweet foods, an intake that can be modulated with exposure (Sclafani, 2006) associated to the energy that the sugars provide (McCaughey, 2008). The increase in sucrose (energy) ingestion may contribute to increase fat deposition, since fructose has been recognized as highly obesogenic (Bocarsly et al., 2010). Fructose (largely as sucrose) is widely present in many Western diets and can induce obesity, including prenatal obesity (Szostaczuk et al., 2017). In rodents, a sucrose-rich diet can rapidly induce a pathologic condition comparable to human metabolic syndrome (Santuré et al., 2002). We assume that the effect of sweet taste may complement the *flavour* of fat texture, in K, despite fatty acids with more powerful “fat taste” not being directly available (Strik et al., 2010).

Rats, like humans, prefer to drink sweet or salty solutions rather than plain water (Khavari, 1970). We can add that salt is known for its taste-enhancing properties, thus increasing the taste effects of all diet components, as well as a reward response, since preferences for both, sweet and salty tastes are mediated by endogenous opioids (Nascimento et al., 2012). In fact, the contrast sweet/salty is one of the key factors establishing the powerful drive to eat, elicited (in humans, at least) by varied food offerings (Low, Lacy & Keast, 2014), thus the factor “variety” could largely be correlated with the presence of these main ancestral sought-for tastes (Naim et al., 1985). Sweets are the most classical “comfort food” (Rho et al., 2014). In humans this slot is covered largely by sweet chocolate, but previous experiments showed that rats do not like the bitter taste of chocolate (Prats et al., 1989), thus sugared milk may be a very good substitute.

Sodium is an essential element actively sought and massively consumed by animals (and evidently including humans) when found (Dahl, 1958), hence our evolutionary drive to consume salt in excess (Morris, Na & Johnson, 2008). The maintenance of normal plasma protein levels suggest limited, if any, effects of high salt intake on the rat water balance, as previously found (Fernández-López et al., 1994). Despite these antecedents, salt intake has not been described as an essential factor eliciting hyperphagia of cafeteria diets. In the case of humans, it is almost impossible to avoid even minimal amounts of salt in present-day diets, whereas its presence in foods akin to cafeteria diets points to a relevant role in the hyperphagia. Furthermore, the effects of salt intake on the renin-angiotensin system (Drenjancevic-Peric et al., 2011), and their effect on corticosteroid secretion along the corticosterone-aldosterone axis have seldom been taken into account in this context. We can speculate that the increased secretion of corticoids as a response to salt (Lewicka, Nowicki & Vecsei, 1998) may help elicit metabolic changes that favour the development of the conditions driving to metabolic syndrome (Alemany, 2012), and the consequent increased lipid deposition (Moosavian et al., 2017).

There were distinctive differences between sexes in taste preferences when the rats were allowed to select foods, as is the case of cafeteria diets. Female rats ingested almost 40% more salt than males when the intake was expressed with respect to body weight increase. These data confirm that female rats show a higher preference for salt than males (Flynn, Schulkin & Havens, 1993). Furthermore, female rats also ingested more sugar, either in absolute or in relative values (i.e., g ingested per g of body weight increase) than males. The preferences of female rats for these nutrients, however, did not result in increased weight, in part because of their higher energy expenditure (Rodríguez-Cuenca et al., 2002) even after correction of size by an allometric factor (Nair & Jacob, 2016). These sex differences could be traced to sex-specific factors of architecture and maturation of the reward system (Gugusheff, Ong & Muhlhausler, 2015). In this context, we have no data about the implication of lactose in taste, although it is well known that the intake of milk (for its taste) also implies the consumption of other milk components, such as active peptides and oestrone (García-Peláez et al., 2004) responsible for higher efficiency in energy deposition during lactation. Furthermore, female rats showed lower increases in circulating triacylglycerols, and lower urea levels than males, in agreement with previous reports (Agnelli et al., 2016), being largely “protected” from excess fat deposition by oestrogens (Zhu et al., 2013).

In this study we assumed that the contribution of protein taste (umami) to food consumption increase can be considered minimal, since the presence of protein (and its quality) was similar (and more than enough in quantities) in all diets; but essentially because dietary protein limits food intake (Anderson & Moore, 2004) in part due to its high satiating effect (Bensaid et al., 2002). The possible effect of protein on food intake in HF groups was, probably, of limited extent, since it was the same (albeit partially diluted) protein of the control diet, and the lack of differences C *vs.* HF prove that they did not act as a differential inductor of satiety as in other models (Bensaid et al., 2002). Conversely, the higher intake of protein in cafeteria groups should elicit a higher satiating effect of proteins; opposing, in fact, the combined actions of sugar and salt (and fat taste) inducing higher food intake. The balance of these opposing effects did not support a significant role of protein in the control of food intake in this model, being superseded by the hedonic influence of more intense tastes (sweet-salty) of food. To our knowledge, no effects of salt enhancing the properties on amino acid and umami taste has been described, so far, in rats (Kurihara, 2015).

## Conclusions

The data presented confirm the higher taste-induced appetite of rats for cafeteria diets, which we can also describe as multichoice high-fat, high-sugar and high-salt compared with most high-fat diets. The higher overall energy intake, in part a consequence of the attenuated satiation mechanisms, the increased variety of food items, and the comfort-food effect (the latter—probably largely as a consequence of the admixture and abundance of sweet-salty taste of food items) enhance the effect of the cafeteria diet to rapidly increase body energy stores. These combined actions favour the development of metabolic syndrome. The perils linked to cafeteria diets are not, thus, limited to high dietary fat content and energy density, but largely to a powerful hedonic component (taste) which can effectively override the normal mechanisms, controlling food (energy) intake.

## Additional Information and Declarations

### Data Availability

The following information was supplied regarding data availability: University of Barcelona institutional repository:
http://hdl.handle.net/2445/111074.

## References

[ref-1] Adam CL, Gratz SW, Peinado DI, Thompson LM, Garden KE, Williams PA, Richardson AJ, Ross AW (2016). Effects of dietary fibre (pectin) and/or increased protein (casein or pea) on satiety, body weight, adiposity and caecal fermentation in high fat diet-induced obese rats. PLOS ONE.

[ref-2] Agnelli S, Arriarán S, Oliva L, Remesar X, Fernández-López J-A, Alemany M (2016). Modulation of rat liver urea cycle and related ammonium metabolism by sex and cafeteria diet. RSC Advances.

[ref-3] Alemany M (2012). Do the interactions between glucocorticoids and sex hormones regulate the development of the metabolic syndrome?. Frontiers in Endocrinology.

[ref-4] Anderson GH, Moore SE (2004). Dietary proteins in the regulation of food intake and body weight in humans. Journal of Nutrition.

[ref-5] Archer ZA, Corneloup J, Rayner DV, Barrett P, Moar KM, Mercer JG (2007). Solid and liquid obesogenic diets induce obesity and counter-regulatory changes in hypothalamic gene expression in juvenile Sprague-Dawley rats. Journal of Nutrition.

[ref-6] Bensaid A, Tome D, Gietzen D, Even P, Morens C, Gausseres N, Fromentin G (2002). Protein is more potent than carbohydrate for reducing appetite in rats. Physiology & Behaviour.

[ref-7] Bocarsly ME, Powell ES, Avena NM, Hoebel BG (2010). High-fructose corn syrup causes characteristics of obesity in rats: increased body weight, body fat and triglyceride levels. Pharmacology Biochemistry and Behaviour.

[ref-8] Breslin PA, Spector AC, Grill HJ (1995). Sodium specificity of salt appetite in Fischer-344 and Wistar rats is impaired by chorda tympani nerve transection. American Journal of Physiology.

[ref-9] Briaud I, Kelpe CL, Johnson LM, Tran PO, Poitut V (2002). Differential effects of hyperlipidemia on insulin secretion in islets of Langerhans from hyperglycemic versus normoglycemic rats. Diabetes.

[ref-10] Buettner R, Parhofer KG, Woenckhaus M, Wrede CE, Kunz-Schugart LA, Schölmerich, Bollheimer LC (2006). Defining high-fat-diet rat models: metabolic and molecular effects of different fat types. Journal of Molecular Endocrinology.

[ref-11] Buettner R, Schölmerich J, Bollheimer LC (2007). High-fat diets: modeling the metabolic disorders of human obesity in rodents. Obesity.

[ref-12] Crescenzo R, Bianco F, Mazzoli A, Giacco A, Cancelliere R, Di Fabio G, Zarrelli A, Liverini G, Iossa S (2015). Fat quality influences the obesogenic effect of high fat diets. Nutrients.

[ref-13] Dahl LK (1958). Salt intake and salt need. New England Journal of Medicine.

[ref-14] Drenjancevic-Peric I, Jelakovic B, Lombard JH, Kunert MP, Kibel A, Gros M (2011). High-salt diet and hypertension: focus on the renin-angiotensin system. Kidney Blood Pressure Research.

[ref-15] Ellis J, Lake A, Hoover-Plow J (2002). Monounsaturated canola oil reduces fat deposition in growing female rats fed a high or low fat diet. Nutrition Research.

[ref-16] Esteve M, Rafecas I, Fernández-López JA, Remesar X, Alemany M (1992a). Fatty acid utilization by young Wistar rats fed a cafeteria diet. Molecular and Cellular Biochemistry.

[ref-17] Esteve M, Rafecas I, Remesar X, Alemany M (1992b). Nitrogen balance of lean and obese Zucker rats subjected to a cafeteria diet. International Journal of Obesity.

[ref-18] Faturi CB, Leite JR, Alves PB, Canton AC, Teixeira-Silva F (2010). Anxiolytic-like effect of sweet orange aroma in Wistar rats. Progress in Neuropsychopharmacol & Biological Psychiatry.

[ref-19] Fernández-López J, Rafecas I, Esteve M, Remesar X, Alemany M (1994). Effect of genetic and dietary obesity on sodium, potassium, calcium and magnesium handling by the rat. International Journal of Food Science and Nutrition.

[ref-20] Flynn FW, Schulkin J, Havens M (1993). Sex differences in salt preference and taste reactivity in rats. Brain Research Bulletin.

[ref-21] Folch J, Lees M, Sloane-Stanley GH (1957). A simple method for the isolation and purification of total lipides from animal tissues. Journal of Biological Chemistry.

[ref-22] García-Peláez B, Ferrer-Lorente R, Gómez-Ollés S, Fernández-López JA, Remesar X, Alemany M (2004). A method for the measurement of estrone content in foods. Application to dairy products. Journal of Dairy Science.

[ref-23] Gomez-Smith M, Karthikeyan S, Jeffers MS, Janik R, Thomason LA, Stefanovic B, Corbett D (2016). A physiological characterization of the Cafeteria diet model of metabolic syndrome in the rat. Physiology & Behavior.

[ref-24] Gugusheff JR, Ong ZY, Muhlhausler BS (2015). The early origins of food preferences: targeting the critical windows of development. FASEB Journal.

[ref-25] Hamilton CL (1964). Rat’s preference for high fat diets. Journal of Comparative Physiological Psychology.

[ref-26] Hariri N, Thibault L (2010). High-fat diet-induced obesity in animal models. Nutrition Research Reviews.

[ref-27] Johnson AR, Wilkerson MD, Sampey BP, Troester MA, Hayes DN, Makowski L (2016). Cafeteria diet-induced obesity causes oxidative damage in white adipose. Biochemical and Biophysical Research Communications.

[ref-28] Kanarek RB, Marks-Kaufman R (1979). Developmental aspects of sucrose-induced obesity in rats. Physiology & Behaviour.

[ref-29] Kant AK, Andon MB, Angelopoulus TJ, Rippe JM (2008). Association of breakfast energy density with diet quality and body mass index in American adults: national health and nutrition examination surveys, 1999–2004. American Journal of Clinical Nutrition.

[ref-30] Khavari KA (1970). Some parameters of sucrose and saline ingestion. Physiology & Behaviour.

[ref-31] Kurihara K (2015). Umami the fifth basic taste: history of studies on receptor mechanisms and role as a food flavor. BioMed Research International.

[ref-32] Lewicka S, Nowicki M, Vecsei P (1998). Effect of sodium on urinary excretion of cortisol and its metabolites in humans. Steroids.

[ref-33] Lladó I, Picó C, Palou A, Pons A (1995). Protein and amino acid intake in cafeteria fed obese rats. Physiology & Behaviour.

[ref-34] Low YQ, Lacy K, Keast R (2014). The role of sweet taste in satiation and satiety. Nutrients.

[ref-35] Lowry OH, Rosebrough RW, Farr AL, Randall RJ (1951). Protein measurement with the Folin phenol reagent. Journal of Biological Chemistry.

[ref-36] Martire SI, Holmes N, Westbrook RF, Morris MJ (2013). Altered feeding patterns in rats exposed to a palatable cafeteria diet: increased snacking and its implications for development of obesity. PLOS ONE.

[ref-37] McCaughey SA (2008). The taste of sugars. Neuroscience & Biobehavioral Reviews.

[ref-38] Miwa I, Maeda K, Okuda J, Okuda G (1972). Mutarotase effect on colorimetric determination of blood-glucose with b-D-glucose oxidase. Clinica Chimica Acta.

[ref-39] Mizushige T, Inoue K, Fushiki T (2007). Why is fat so tasty? Chemical reception of fatty acid on the tongue. Journal of Nutritional Science and Vitaminology.

[ref-40] Moore BJ (1987). The cafeteria diet. An inappropriate tool for studies of thermogenesis. Journal of Nutrition.

[ref-41] Moore CJ, Michopoulos V, Johnson ZP, Toufexis D, Wilson ME (2013). Dietary variety is associated with larger meals in female rhesus monkeys. Physiology & Behavior.

[ref-42] Moosavian SP, Haghighatdoost F, Surkan PJ, Azadbakht L (2017). Salt and obesity: a systematic review and meta-analysis of observational studies. International Journal of Food Sciences and Nutrition.

[ref-43] Morris MJ, Na ES, Johnson AK (2008). Salt craving: the psychobiology of pathogenic sodium intake. Physiology & Behaviour.

[ref-44] Mrosovsky N, Powley TL (1977). Set points for body weight and fat. Behaviour Biologica.

[ref-45] Muntzel MS, Al-Naimi OAS, Barclay A, Ajasin D (2012). Cafeteria diet increases fat mass and chronically elevates lumbar sympathetic nerve activity in rats. Hypertension.

[ref-46] Naim M, Brand JG, Kare MR, Carpenter RG (1985). Energy intake, weight gain and fat deposition in rats fed flavored, nutritionally controlled diets in a multichoice (“cafeteria”) design. Journal of Nutrition.

[ref-47] Nair AB, Jacob S (2016). A simple practice guide for dose conversion between animals and human. Journal of Basic Clinical Pharmacy.

[ref-48] Nascimento AIR, Ferreira HS, Saraiva RM, Almeida TS, Fregoneze JB (2012). Central kappa opioid receptors modulate salt appetite in rats. Physiology & Behaviour.

[ref-49] Oakes ND, Cooney GJ, Camilleri S, Chisholm DJ, Kraegen EW (1997). Mechanisms of liver and muscle insulin resistance induced by chronic high-fat feeding. Diabetes.

[ref-50] Oliva L, Baron C, Fernández-López J-A, Remesar X, Alemany M (2015). Marked increase in rat red blood cell membrane protein glycosylation by one-month treatment with a cafeteria diet. PeerJ.

[ref-51] Ortolani D, Oyama LM, Ferrari EM, Melo LL, Spadari-Bratfisch RC (2011). Effects of comfort food on food intake, anxiety-like behavior and the stress response in rats. Physiology & Behavior.

[ref-52] Peciña S, Smith KS, Berridge KC (2006). Hedonic hot spots in the brain. The Neuroscientist.

[ref-53] Peckham SC, Entenman C, Carroll HW (1977). The influence of a hypercaloric diet on gross body and adipose tissue composition in the rat. Journal of Nutrition.

[ref-54] Pini RTB, Do Vales LDMF, Braga Costa TM, Almeida SS (2016). Effects of cafeteria diet and high fat diet intake on anxiety, learning and memory in adult male rats. Nutritional Neuroscience.

[ref-55] Portillo MP, Serra F, Simon E, Del Barrio AS, Palou A (1998). Energy restriction with high-fat diet enriched with coconut oil gives higher UCP1 and lower white fat in rats. International Journal of Obesity.

[ref-56] Prats E, Monfar M, Iglesias R, Castellà J, Alemany M (1989). Energy intake of rats fed a cafeteria diet. Physiology & Behavior.

[ref-57] Radcliffe J, Webster A (1976). Regulation of food intake during growth in fatty and lean female Zucker rats given diets of different protein content. British Journal of Nutrition.

[ref-58] Rafecas I, Esteve M, Fernández-López JA, Remesar X, Alemany M (1992). Deposition of dietary fatty acids in young Zucker rats fed a cafeteria diet. International Journal of Obesity.

[ref-59] Rafecas I, Esteve M, Fernández-López JA, Remesar X, Alemany M (1993). Individual amino acid balances in young lean and obese Zucker rats fed a cafeteria diet. Molecular and Cellular Biochemistry.

[ref-60] Ramirez I, Friedman MI (1990). Dietary hyperphagia in rats—role of fat, carbohydrate, and energy content. Physiology & Behaviour.

[ref-61] Reichelt AC, Morris MJ, Westbrook R (2014). Cafeteria diet impairs expression of sensory-specific satiety and stimulus-outcome learning. Frontiers in Psychology.

[ref-62] Rho SG, Kim YS, Choi SC, Lee MY (2014). Sweet food improves chronic stress-induced irritable bowel syndrome-like symptoms in rats. World Journal of Gastroenterology.

[ref-63] Rodríguez-Cuenca S, Pujol E, Justo R, Fronteras M, Oliver J, Gianotti M, Roca P (2002). Sex-dependent thermogenesis, differences in mitochondrial morphology and function, and adrenergic response in brown adipose tissue. Journal of Biological Chemistry.

[ref-64] Romero MM, Holmgren F, Grasa MM, Esteve M, Remesar X, Fernández-López JA, Alemany M (2013). Modulation in Wistar rats of blood corticosterone compartmentation by sex and prior exposure to a cafeteria diet. PLOS ONE.

[ref-65] Romero MM, Roy S, Pouillot K, Feito M, Esteve M, Grasa MM, Fernández-López JA, Remesar X, Alemany M (2014). Treatment of rats with a self-selected hyperlipidic diet, increases the lipid content of the main adipose tissue sites in a proportion similar to that of the rest of body lipid stores. PLOS ONE.

[ref-66] Rothwell NJ, Saville ME, Stock MJ (1982). Effects of feeding a “cafeteria” diet on energy balance and diet-induced thermogenesis in four strains of rat. Journal of Nutrition.

[ref-67] Rothwell NJ, Stock MJ (1984). The development of obesity in animals: the role of dietary factors. Clinical Endocrinology and Metabolism.

[ref-68] Santuré M, Pitre M, Marette A, Deshaies Y, Lemieux C, Larivière R, Nadeau A, Bachelard H (2002). Induction of insulin resistance by high-sucrose feeding does not raise mean arterial blood pressure but impairs haemodynamic responses to insulin in rats. British Journal of Pharmacology.

[ref-69] Sato A, Kawano H, Notsu T, Ohta M, Nakakuki M, Mizuguchi K, Itoh M, Suganami T, Ogawa Y (2010). Antiobesity effect of eicosapentaenoic acid in high-fat/high-sucrose diet—induced obesity. Importance of hepatic lipogenesis. Diabetes.

[ref-70] Scharrer E (1999). Control of food intake by fatty acid oxidation and ketogenesis. Nutrition.

[ref-71] Schemmel R, Mickelsen O, Tolgay Z (1969). Dietary obesity in rats: influence of diet, weight, age, and sex on body composition. American Journal of Physiology.

[ref-72] Sclafani A (1987). Carbohydrate-induced hyperphagia and obesity in the rat: effects of saccharide type, form, and taste. Neuroscience & Biobehavioral Reviews.

[ref-73] Sclafani A (2006). Enhanced sucrose and Polycose preference in sweet “sensitive”(C57BL/6J) and “subsensitive”(129P3/J) mice after experience with these saccharides. Physiology & Behaviour.

[ref-74] Sclafani A, Gorman AN (1977). Effects of age, sex, and prior body weight on the development of dietary obesity in adult rats. Physiology & Behavior.

[ref-75] Sclafani A, Springer D (1976). Dietary obesity in adult rats: similarities to hypothalamic and human obesity syndromes. Physiology & Behavior.

[ref-76] Strik CM, Lithander FE, McGill AT, MacGibbon AK, McArdle BH, Poppitt SD (2010). No evidence of differential effects of SFA, MUFA or PUFA on post-ingestive satiety and energy intake: a randomised trial of fatty acid saturation. Nutrition Journal.

[ref-77] Szostaczuk N, Priego T, Palou M, Palou A, Picó C (2017). Oral leptin supplementation throughout lactation in rats prevents later metabolic alterations caused by gestational calorie restriction. International Journal of Obesity.

[ref-78] Tordoff MG, Reed DR (1991). Sham-feeding sucrose or corn oil stimulates food intake in rats. Appetite.

[ref-79] Zeeni N, Bassil M, Fromentin G, Chaumontet C, Darcel N, Tome D, Daher CF (2015). Environmental enrichment and cafeteria diet attenuate the response to chronic variable stress in rats. Physiology & Behavior.

[ref-80] Zhu L, Brown WC, Cai Q, Krust A, Chambon P, McGuiness OP, Stafford JM (2013). Estrogen treatment after ovariectomy protects against fatty liver and may improve pathway-selective insulin resistance. Diabetes.

